# Development and verification of a discriminate algorithm for diagnosing post‐neurosurgical bacterial meningitis—A multicenter observational study

**DOI:** 10.1002/jcla.23069

**Published:** 2019-10-10

**Authors:** Guanghui Zheng, Xufeng Ji, Xiaochen Yu, Min Liu, Jing Huang, Lina Zhang, Dawen Guo, Guojun Zhang

**Affiliations:** ^1^ Department of Clinical Diagnosis Laboratory of Beijing Tiantan Hospital and Capital Medical University Beijing China; ^2^ Department of Clinical Diagnosis Laboratory of the First Hospital of Jilin University Changchun China; ^3^ Laboratory Diagnosis Department of the Affiliated Hospital of Harbin Medical University Harbin China; ^4^ Daqing Oilfield General Hospital Clinical Laboratory Daqing China

**Keywords:** algorithm, cerebrospinal fluid, PNBM, receiver operating characteristic

## Abstract

**Objective:**

To evaluate the diagnostic accuracy of cerebrospinal fluid (CSF)–based routine clinical examinations for post‐neurosurgical bacterial meningitis (PNBM) in multicenter post‐neurosurgical patients.

**Methods:**

The diagnostic accuracies of routine examinations to distinguish between PNBM and post‐neurosurgical aseptic meningitis (PNAM) were evaluated by determining the values of the area under the curve (AUC) of the receiver operating characteristic curve in a retrospective analysis of post‐neurosurgical patients in four centers.

**Results:**

An algorithm was constructed using the logistic analysis as a classical method to maximize the capacity for differentiating the two classes by integrating the measurements of five variables. The AUC value of this algorithm was 0.907, which was significantly higher than those of individual routine blood/CSF examinations. The predicted value from 70 PNBM patients was greater than the cutoff value, and the diagnostic accuracy rate was 75.3%. The results of 181 patients with PNAM showed that 172 patients could be correctly identified with specificity of 95.3%, while the overall correctness rate of the algorithm was 88.6%.

**Conclusions:**

Routine biomarkers such as CSF/blood glucose ratio (C/B‐Glu), CSF lactate (C‐Lac), CSF glucose concentration (C‐Glu), CSF leukocyte count (C‐Leu), and blood glucose concentration (B‐Glu) can be used for auxiliary diagnosis of PNBM. The multicenter retrospective research revealed that the combination of the five abovementioned biomarkers can effectively improve the efficacy of the PNBM diagnosis.

## 1 INTRODUCTION

Post‐neurosurgical meningitis (PNM), a common complication of neurosurgical procedures, has an incidence rate of 0.3%‐25%.^1^ PNM significantly affects the length of hospital stay (LOS), increases the cost, decreases the success rate of neurosurgery, and increases patients’ mortality rate. PNM can cause approximately 35% mortality.^2^ PNM has been mainly divided into post‐neurosurgical bacterial meningitis (PNBM) and post‐neurosurgical aseptic meningitis (PNAM).^3^ The pathogenesis and treatment for PNBM and PNAM are completely different. The main cause of PNBM is infections induced by pathogenic bacteria; the onset of PNBM is rapid, and antibiotics are required for treatment. Scattered bone fragments or tumor antigens produced during neurosurgery may be the cause of PNAM, and no antibiotics are required for its treatment.^4^ PNBM and PNAM always share some physical signs and clinical symptoms, including mental status changes, neck stiffness, headache, fever, and vomiting, which do not sufficiently specify the clinical manifestations of both meningitis forms to diagnose PNBM.^5^ Given these influencing factors, rapid diagnostic methods are needed urgently to provide more diagnostic options and evaluate the efficacy of antibiotic drugs.

Presently, regardless of the Infectious Diseases Society of America (IDSA) 6 and Centres for Disease Control and Prevention (CDC)^7^ criteria, the diagnostic strategy for PNBM is based on the combination of clinical symptoms and laboratory tests of the patients. The gold diagnosis standard for bacterial meningitis is the cerebrospinal fluid (CSF) bacterial culture. Culture is, however, unreliable due to time consumption and extensive antibiotic prophylaxis ahead of neurosurgical operations, and the results are available only after a couple of days. Several novel biomolecular approaches to the rapid diagnosis of PNBM have been applied in clinical laboratories, such as PCR and next‐generation sequencing (NGS). However, these new approaches have some specific weaknesses. PCR has a higher false‐positive feature and does not meet the need. On the other hand, although NGS has high‐throughput properties, the cost, complexity, and time consumption of NGS are not suitable for routine clinical application.^8^ With the help of multivariable diagnostic strategy, parallelly combined routine CSF or blood laboratory tests have become one of the options for the clinical laboratory diagnosis of PNM. However, large‐scale clinical studies and laboratory data on the diagnosis of PNBM are still lacking, and consequently, parallelly combined routine CSF or blood laboratory variable detection in multicenters managing PNM is a matter of cardinal significance.

Here, to optimize the PNBM diagnosis, we established two cohorts in this study. Through the first cohort, we retrospectively evaluated 14 routine CSF/blood infection‐related biomarkers in the cases of PNBM collected during 2012‐2016 at Beijing Tiantan Hospital and Capital Medical University, and we then constructed an algorithm for the diagnosis of PNBM. After that, the related biomarkers from the patients with PNBM in four tertiary hospitals in northern China acted as the validation cohort to evaluate the algorithm.

## 2 MATERIALS AND METHODS

### 2.1 Study design and data abstraction

This study was first performed on patients suspected to have PNM and hospitalized during 2012‐2018 at Beijing Tiantan Hospital, a tertiary hospital with 13 000 neurosurgical operations conducted annually. All the patients who underwent post‐neurosurgery during this period were eligible for this study. The variables were retrospectively obtained from the databases of clinical laboratory information system, and 14 clinical laboratory variables related to PNM were initially reviewed in this study, including CSF cell count (C‐Cell), CSF leukocyte count (C‐Leu), CSF neutrophil proportions (C‐Neu), chloride ion concentration (B‐Cl‐), CSF protein concentration (C‐Pro), CSF glucose concentration (C‐Glu), CSF lactate (C‐Lac), blood glucose concentration (B‐Glu), CSF/blood glucose ratio (C/B‐Glu), blood leukocyte count (B‐Leu), blood neutrophil proportions (B‐Neu), red blood cell count (RBC), hemoglobin concentration (Hb), and platelet (PLT) count. This study was approved by the clinical laboratory of Beijing Tiantan Hospital and Capital Medical University. As this study did not refer to the individual information of the patients, no specific ethical consent was obtained for this study.

### 2.2 Definition of neurosurgical meningitis

#### 2.2.1 Inclusion and exclusion criteria

This study performed as a gold standard for the diagnosis of meningitis in groups of PNBM and PNAM.^9^ The inclusion criteria were as follows: (a) clinical manifestations of neurological infections (body temperature >38.3°C, headache, neck stiffness, etc), (b) positive CSF culture, and (c) antibiotic treatment is effective. The meningitis was considered as PNBM when all three criteria were met. The PAM patients required a diagnostic lumbar puncture, but did not receive postoperative antibiotic treatments. The specific criteria are as follows: (a) abnormalities in laboratory tests related to CSF and (b) diagnostic criteria for bacterial meningitis not met.^10^ Patients who were immunocompromised and had CSF shunt infections, had coagulase‐negative staphylococci infection, or showed the presence of cryptococcal antigen, intracranial masses, and brain abscesses were excluded from the study.^11^

#### 2.2.2 Specimen collection

The CSF samples of all the patients were collected on the day when the body temperature exceeded 38.3°C. CSF specimens were obtained by lumbar puncture, lumbar cistern drainage, ventricular drainage, or ventriculoscopy. Blood specimens were obtained by the conventional venous blood collection method.

### 2.3 Statistical methods

All the variables were analyzed using SPSS software version 20 (IBM).

Continuous data were expressed as mean ± SD or median (25%, 75%), whereas categorical data were expressed as numbers and percentages. Continuous variables were analyzed by nonparametric Mann‐Whitney *U* test or Student's *t* test when appropriate, and chi‐square or Fisher's exact test was performed for the categorical data. Univariate analysis was employed to calculate the *P* values for all variables; a multivariate algorithm was performed to take into account differences between the two groups by using a logistic regression. All the variables whose *P* was < .05 were embedded into the one fitting variable.^12^ Diagnosis accuracy of the fitted variable was evaluated by area under the curve (AUC) of the receiver operating characteristic (ROC) curve analyses, and the sensitivity, specificity, positive predictive value (PPV), negative predictive value (NPV), Youden score, and cutoff values were also calculated. The established algorithm was validated by the data of the patients at four neurosurgical centers.

## 3 RESULTS

### 3.1 Patient characteristics and microbiological results of the samples

During the first step of this study, 12 025 patients were recruited; of them, a total of 226 patients with PNBM and 255 with PNAM were enrolled in the study. The median age of this cohort was 51.8 years with a minimum of 18 years and a maximum of 84 years. In 226 cases of PNBM, Klebsiella pneumonia, Acinetobacter baumannii, Staphylococcus aureus, Enterococcus faecalis, and Escherichia coli were the five most common pathogens isolated from patients’ samples. The distribution of pathogens that caused PNBM is shown in Table 1.

**Table 1:** Numbers and proportion of pathogens that caused PNBM in Beijing Tiantan Hospital

Bacteria	Numbers	Proportion (%)
*Klebsiella pneumoniae*	38	16.89
*Acinetobacter baumannii*	35	15.56
*Staphylococcus aureus*	22	9.78
*Enterococcus faecalis*	20	8.89
*Escherichia coli*	16	7.11
*Enterococcus faecium*	13	5.78
*Pseudomonas aeruginosa*	12	5.33
*Klebsiella oxytoca*	8	3.56
*Enterobacter cloacae*	7	3.11
Others	54	24.00

### 3.2 Univariate analysis of the parameters

We evaluated the diagnostic accuracy of these variables with a statistically significant difference. The result of the univariate analysis for each variable is shown in Table 2. Among these tests, in general, CSF variables performed better than blood variables. All the CSF biomarkers, including C‐Cell (10^6^/L), C‐Leu (10^6^/L), C‐Neu (%), C‐Glu (mmol/L), C‐Pro (mg/dL), C‐Cl^‐^ (mmol/L), and B‐Glu (mmol/L), showed a statistically significant difference between the PNBM group and the PNAM group.

**Table 2:** Univariate analysis of the parameters of the PNBM and PNAM patients

Variable	PNBM (226)	PNAM (255)	*P*
Age (years)	48 (37, 59)	45 (35, 56)	.814
Male (%)	121 (53.5)	136 (53.3)	.971
CSF biomarkers			
C‐Cell (10^6^/L)	6529.0 (2107.0, 22 714.0)	2509.0 (214.0, 22 741.0)	<.001
C‐Leu (10^6^/L)	2064.0 (716.5, 7399.3)	73.0 (10.0, 3060.0)	<.001
C‐Neu (%)	88.1 (80.5, 95.1)	38.4 (0.0, 91.9)	<.001
C‐Glu (mmol/L)	1.6 (0.78, 2.80)	3.4 (2.6, 4.3)	<.001
C‐Pro (mg/dL)	180.0 (98.0, 293.8)	89.8 (42.8, 220.1)	<.001
C‐Cl^‐^ (mmol/L)	117.0 (111.9, 122.0)	121.1(116.6, 124.8)	<.001
C/B‐Glu	0.25 (0.08, 0.44)	0.64(0.45, 0.84)	<.001
C‐Lac (μmol/L)	5.1 (3.5, 8.0)	2.7 (1.9, 4.2)	<.001
Blood biomarkers			
B‐Glu (mmol/L)	6.6 (5.4, 9.0)	5.0 (4.3, 6.4)	<.001
B‐Leu (10^9^/L)	12.4 (9.4, 17.4)	13.5 (9.5, 17.1)	.616
B‐Neu (%)	85.0 (77.2, 89.6)	86.1 (75.6, 90.7)	.872
RBC (10^12^/L)	3.8 ± 0.7	4.1 ± 0.7	<.001
Hb (g/L)	114.2 ± 22.1	120.3 ± 21.0	.002
PLT (10^9^/L)	230.5 (188.8, 286.0)	236.0 (190.0, 295.0)	.738

Note

*P* < .05 was considered to be significant.

### 3.3 Multivariate analysis of the parameters

We included all the factors with *P* value < .2 in the univariate analysis into the logistic multivariate analysis. C‐Leu, C‐Glu, B‐Glu, C/B‐Glu, and C‐Lac were determined as the parameters for the algorithm's construction (Table 3). Simultaneously, an algorithm was constructed with the analysis, which is a classification method that integrated the five abovementioned biomarkers to maximize the capacity for differentiating between the PNBM and PNAM groups. The algorithm of PNBM is as follows: *F* = −0.176*C‐Glu + 0.191*B‐Glu + 0.515*C‐Lac‐3.351*C/B‐Glu + 0.0000173*C‐Leu‐1.584.

**Table 3:** Multivariate analysis of the parameters of the PNBM and PNAM patients

Variable	B	*P*	OR	95% confidence interval
C‐Glu	−0.176	.034	1.192	0.64‐2.222
B‐Glu	0.191	.046	0.826	0.643‐1.062
C/B‐Glu	−3.351	<.001	28.533	0.723‐1126.799
C‐Lac	0.515	<.001	0.597	0.517‐0.691
C‐Leu	1.173E‐05	.036	1.000	1.000‐1.000
Const	−1.584	.04	‐	‐

### 3.4 Verification of the multivariate algorithm

**Figure 1:**
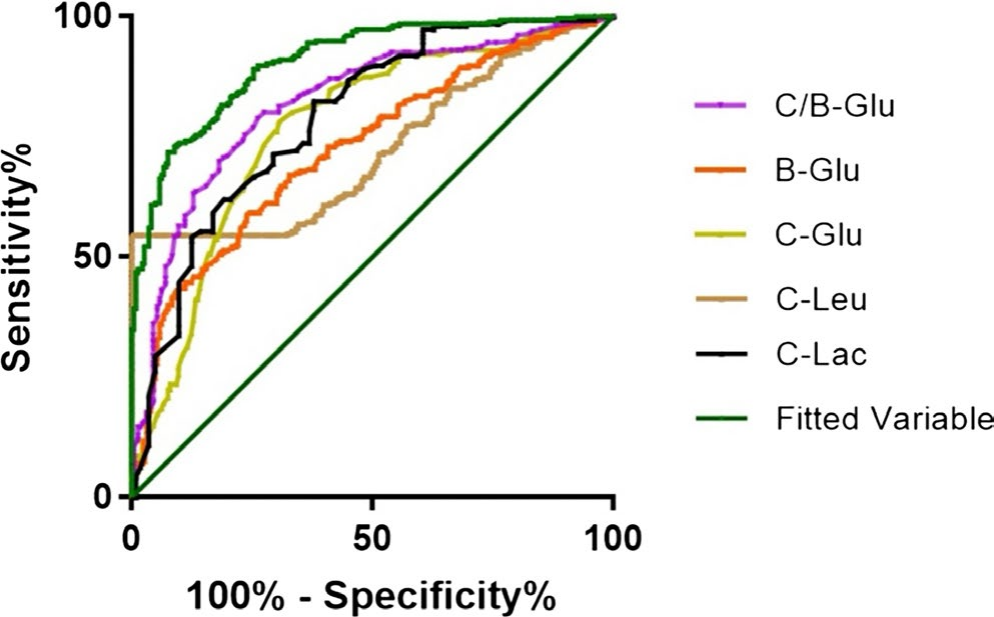
ROC curves of the five parameters and fitted variable of the algorithm

The fitted variable and the five biomarkers above, including C‐Leu (0.754), C‐Glu (0.767), B‐Glu (0.725), C/B‐Glu (0.820), and C‐Lac (0.791), were chosen as the candidate variables for the algorithm's verification (Table 4 and Figure 1). Among these biomarkers, C‐Leu at a cutoff value of 577.5*10^6^/L had the highest specificity (83.6%), and C‐Lac had the best sensitivity (82.4%) and PPV (78.0%). The results of the ROC curve of the fitted biomarkers, which formed in the multivariate algorithm, are shown in Table 5. The AUC of the fitted variable was 0.907, and the sensitivity, specificity, PPV, and NPV were greater than 80.0%. The cutoff of the fitted variable is 0.505. The AUC indicated that the fitted variable was a much better biomarker for distinguishing PNBM from PNAM.

**Table 4:** ROC parameters of the routine cerebrospinal fluid (CSF)/blood parameters for the diagnosis of PNBM

Variable	AUC	Sensitivity (%)	Specificity (%)	PPV (%)	NPV	Youden score	Cutoff
C/B‐Glu	0.820	78.8	74.3	75.4	77.8	0.531	0.43
C‐Lac (μmol/L)	0.791	62.4	82.4	78.0	68.7	0.448	4.45
C‐Glu (mmol/L)	0.767	78.0	69.5	71.9	76.0	0.475	2.45
C‐Leu (10^6^/L)	0.754	83.6	56.0	65.5	77.3	0.396	577.5
B‐Glu (mmol/L)	0.725	76.1	58.8	64.9	71.1	0.349	5.35
C‐Neu (%)	0.692	98.7	40.1	62.2	96.9	0.389	48.65
C‐Pro (mg/dL)	0.661	87.2	43.9	60.9	77.4	0.311	72.91
C‐Cell (10^6^/L)	0.655	88.1	44.8	61.5	79.0	0.318	1262.5
C‐Cl^−^ (mmol/L)	0.651	72.5	52.2	60.3	65.5	0.247	117.35
RBC (10^12^/L)	0.601	77.3	38.5	55.7	62.9	0.158	3.585
Hb (g/L)	0.582	43.1	71.2	59.9	55.6	0.143	126.5

Abbreviations: NPV, negative predictive value; PPV, positive predictive value.

**Table 5:** ROC parameters of the fitted variable for the diagnosis of PNBM

Variable	AUC	Sensitivity	Specificity	PPV	NPV	Youden score	Cutoff
Fitted variable	0.907	84.3%	80.9%	81.5%	83.7%	0.652	0.505

### 3.5 Verification by the hypothetical cohort of 1000 patients

In reality of the proportion of PNBM and PNAM (1:1.1, 476:524 patients), if the conventional clinical laboratory tests were to be used in a group of 1000 patients with neurosurgical meningitis, then an estimated 501 would have a result, indicating that they got a PNBM and of these, 100 (19.9%) would have PNAM, and the PPV = 80.00%; and an estimated 499 patients would have a result, indicating that they got a PNAM and of these, 75 (15.0%) would actually have PNBM, and the NPV = 85.0%. All of the data above are shown in Table 6.

**Table 6:** Chart of the TP and TN of PNBM diagnosed by the multivariate algorithm

Items	Proportion in reality	PNBM	PNAM	Total
TP/FP	476	401	100	TP + FP = 501 (PPV = 80.0%)
TN/FN	524	75	424	FN + TN = 499 (NPV = 85.0%)
Total	1000	476	524	1000

Note

Proportion of PNBM = 47.6%, sensitivity = 47.6%, specificity = 95.4%.

Abbreviations: FN, false negative; FP, false positive; TN, true negative; TP, true positive.

### 3.6 Multicenter verification of the algorithms

**Figure 2:**
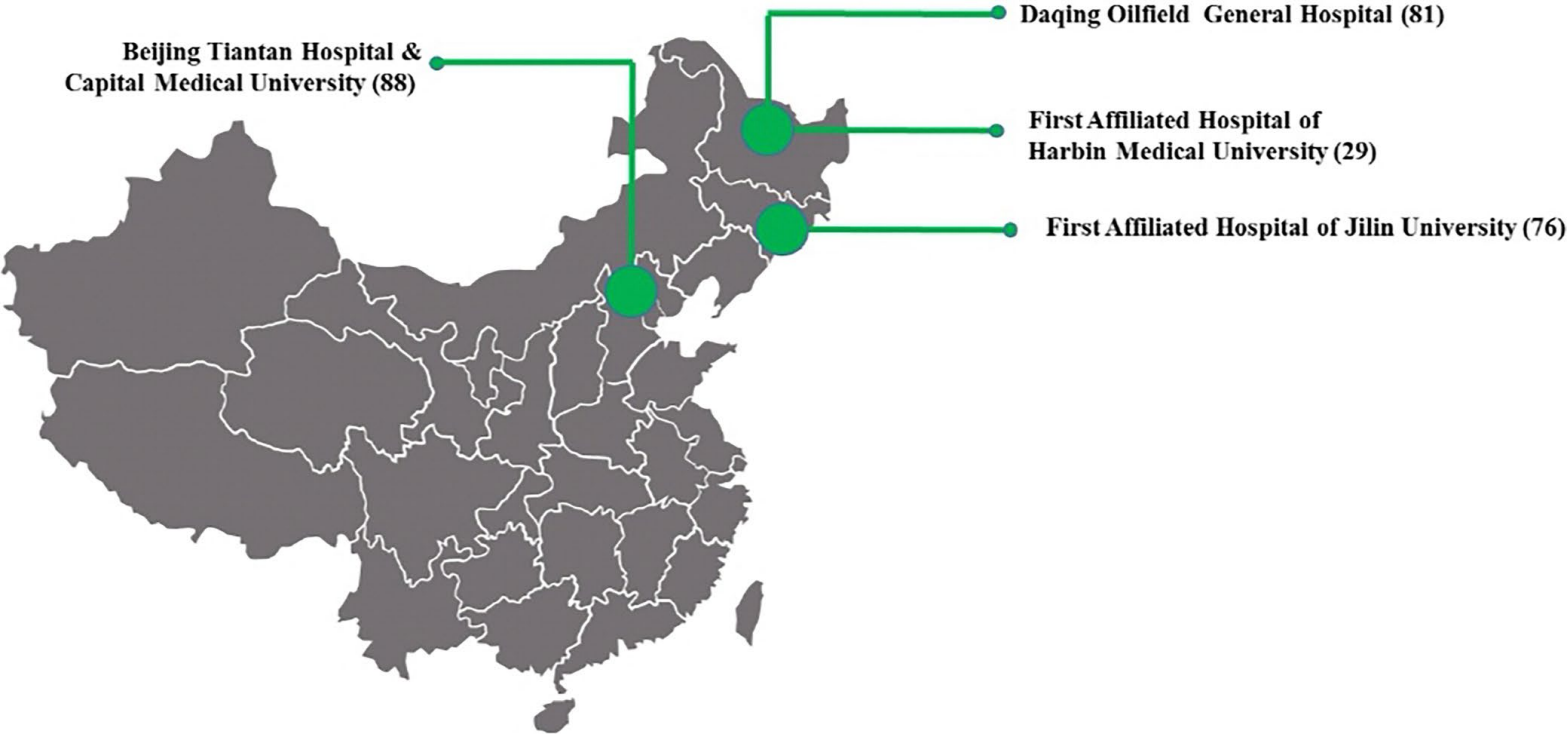
The location of four neurosurgical center PNM patients for the verification of the algorithm

We selected 93 PNBM patients and 181 PNAM patients from four centers in northern China during May‐September 2018 and substituted the values of the biomarkers into the algorithm for differentiating between PNAM and PNBM. There were 29 (7/22, PNBM/PNAM) patients from the First Affiliated Hospital of Harbin Medical University, 76 (11/65, PNBM/PNAM) patients from the First Affiliated Hospital of Jilin University, 81 (28/53, PNBM/PNAM) patients from Daqing Oilfield General Hospital, and 88 (47/41, PNBM/PNAM) patients from Beijing Tiantan Hospital and Capital Medical University. The location of the neurosurgical centers for the diagnosis of PNM is shown in Figure 2.

The algorithm was verified by these 274 confirmed PNM patients from four centers in northern China, and the calculated *F* value was compared with the cutoff (shown in Table 5) value calculated by the ROC curve. Patients whose biomarkers’ values were greater than the 0.505（cutoff value） of the algorithm were classified as PNBM, and patients whose *F* values were less than the 0.505 of the algorithm were classified as PNAM. The results are shown in Table 7. The predicted values of 70 PNBM patients were greater than the cutoff value, and the positive concordance rate was 75.3%. The results of 181 patients with PNAM showed that 172 patients were correctly identified; the negative concordance rate was 95.0%, and the overall concordance rate of the algorithm was 88.6%.

**Table 7:** Evaluation of diagnostic and validation value of four neurosurgical centers between the PNBM and PNAM groups

Neurosurgical centers	Sensitivity (%)	Specificity (%)	PPV (%)	NPV (%)	Concordance rate (%)
First Affiliated Hospital of Harbin Medical University	83.3	91.3	71.4	95.4	89.7
First Affiliated Hospital of Jilin University	72.7	95.4	72.7	95.4	92.1
Daqing Oilfield General Hospital	87.5	87.7	75.0	94.3	86.6
Beijing Tiantan Hospital and Capital Medical University	94.7	78.0	76.6	95.1	85.2
Total	88.6	88.2	75.3	95.0	88.6

## 4 DISCUSSION

PNBM can be diagnosed through clinical symptoms, CSF laboratory biomarker analysis, and Gram staining with some uncertainty, and ideally through CSF bacterial culture. The analysis of CSF laboratory biomarkers is one of the most pivotal methods to diagnose PNBM.^13^ However, many studies have reported that routine infection markers such as CSF or blood biomarkers are not diagnostically optimal due to low specificity,^1^ and therefore, PNBM may not be accurately diagnosed. In our current study, we evaluated the diagnostic accuracy of parallelly combined routine CSF and blood tests for PNBM. An algorithm that can distinguish PNBM from PNAM was established, and a multicenter verification was also performed. The best highlight of this study is that we implemented the most common clinical diagnosis indicators, such as the CSF/blood glucose ratio, lactate, and CSF leukocyte count to make precise diagnosis of PNBM, and our algorithm that passes through verification and confirmation could facilitate delivering a more accurate PNBM diagnosis.

At present, there are many reports on the differentiation between PNBM and PNAM, but almost all the reports on meningitis have included relevant markers, such as C‐Leu, for an auxiliary diagnosis.^4^, ^14^ This grouping method can expand the number of enrolled patients with PNBM, but it is also inevitable to include patients with non‐bacterial meningitis. This study grouped meningitis based on the gold standard of infection and clinical symptoms, which can effectively avoid the inclusion of non‐bacterial meningitis and have higher clinical value.^15^ In this study, we first determined the relevant biomarkers and then performed the algorithm based on the grouping of PNBM and PNAM patients in Beijing Tiantan Hospital and Capital Medical University. Subsequently, verification studies were conducted in four neurosurgical centers in northern China, and a universal algorithm was obtained to provide an auxiliary basis for the diagnosis of PNBM.

CSF and blood biomarkers play an important role in diagnosing meningitis and monitoring the use of antibiotics. Through a retrospective analysis of 14 biomarkers in 481 PNM patients in Beijing Tiantan Hospital, the difference in 11 markers, including eight CSF and three blood markers, in the two groups of patients with meningitis was statistically significant. The AUC showed that the diagnostic efficacy of CSF markers was superior to blood markers. C‐Leu was not the most effective biomarker for the diagnosis of PNBM (AUC = 0.754) because of its high sensitivity (83.6%) and low specificity (56.0%, <60%). Compared with CSF leukocyte–related markers, C‐Glu was more effective in diagnosing PNBM, especially C/B‐Glu, which is the most effective diagnostic indicator of all single markers (AUC = 0.820). The reason may be that there is a consumption of glucose in PNBM, but PNAM patients only experience an inflammatory reaction; thus, the difference between the two is actually significant.^16^ In addition to routine markers, C‐Lac is the second variable for diagnostic efficacy (AUC = 0.791). In a prospective study, C‐Lac can effectively distinguish PNBM from PNAM when its concentration is greater than 6 μmol/L, and the value of C‐Lac was determined to be 4‐6 μmol/L for PNBM treatment period, and <2 μmol/L for PNAM.^17^ Although some biomarkers recommend a good diagnosis ability to discriminate PNBM from PNAM, however, the specificity of the majority mono‐biomarkers was low (eg, C/B‐Glu, 74.30%; C‐Glu, 69.50%; C‐Leu, 56.00%; B‐Glu, 58.80%); thus, the diagnosis of meningitis cannot be performed alone and multiple indicators with combined diagnosis are needed.

Multivariable integrated diagnosis can effectively improve the diagnostic performance with enhanced sensitivity and specificity^18^; hence, we constructed a multivariable algorithm of PNBM. Through the logistic regression analysis, we obtained five biomarkers (C/B‐Glu, C‐Lac, C‐Glu, C‐Leu, and B‐Glu) to find the best compositions and choose them as the candidate to construct algorithm.

The multivariate approach significantly improved the performance of PNBM prediction, and the AUC of the fitted variable was 0.907, which was significantly higher than the values of each individual test; in addition, the specificity and sensitivity were high (80.9% vs 84.3%). An algorithm was constructed using these five biomarkers to differentiate PNBM from PNAM. We recommend *F* = 0.505 in this algorithm as a cutoff to distinguish between PNBM and PNAM in neurosurgical clinical practice, because the sensitivity and specificity are both high. When the value of algorithm was more than cutoff, we should start special antibiotic treatment for PNM and this patient was classified into PNBM (sensitivity, 84.3%; PPV, 81.5%). To the contrary, when the value was <0.505, it is not necessary to start antibiotic treatment immediately, because the possibility for PNAM diagnosis is high (specificity, 80.9%; NPV, 83.7%). Through a hypothesis cohort verification established by the real proportion, we found that the diagnostic ability of the algorithm is also higher than 80%, and only 19.9% and 15.0% of PNBM and PNAM patients have diagnostic bias. The data from 274 patients from four neurosurgical centers in northern China with confirmed PNBM and PNAM were collected for verification and confirmation. The positive and negative concordance rates of the algorithm were high (88.6% and 88.2%). The whole concordance rate was 88.6%, indicating that it is a good algorithm; thus, the use of this algorithm can effectively complete the diagnosis of PNBM.

The main limitation of this study is that it comprised routine examinations, but clinical symptoms such as body temperature and age were not considered. In addition, infection markers such as procalcitonin, interleukin‐6, and C‐reactive protein were not discussed in this study. In our future studies, new biomarkers will be gradually included as variables of the algorithm in order to further increase the sensitivity and specificity of this method.

## 5 CONCLUSION

To summarize, routine biomarkers such as C/B‐Glu, C‐Lac, C‐Glu, C‐Leu, and B‐Glu can be used for the auxiliary diagnosis of PNBM. In this study, multicenter research was performed to show that by combining the five abovementioned biomarkers, we can effectively improve the efficacy of PNBM diagnosis and can speed up the entire diagnostic procedure.

## CONFLICT OF INTEREST

The authors declare no conflict of interest.

## ETHICAL APPROVAL

No ethical approval was needed in this study.
